# Genomic Characterization and Antibiotic Resistance Profiles of *Acinetobacter baumannii* Isolates From Intensive Care Units in Vietnam

**DOI:** 10.1155/ijm/7578951

**Published:** 2025-07-15

**Authors:** Thanh Truc Tran, Phuc Hoang Bui

**Affiliations:** ^1^Department of Biotechnology, Faculty of Chemical Engineering, Ho Chi Minh City University of Technology (HCMUT), VNU-HCM, Ho Chi Minh City, Vietnam; ^2^Faculty of Applied Technology, School of Technology, Van Lang University, Ho Chi Minh City, Vietnam

**Keywords:** carbapenem-resistant *A. baumannii* (CRAB), genomic epidemiology and resistance mechanisms, hospital-acquired infections

## Abstract

*Acinetobacter baumannii*, an opportunistic pathogen, is responsible for a wide range of healthcare-associated infections (HAIs), particularly in patients in intensive care units (ICUs). Carbapenem-resistant *A. baumannii* (CRAB) is of particular concern due to its extensive multidrug resistance (MDR) and limited treatment options. In Vietnam, CRAB has become increasingly prevalent, with resistant mechanisms primarily attributed to the presence of *blaOXA-23* and *blaNDM-1* genes. This study investigates the clinical characteristics and genomic epidemiology of three CRAB isolates (ICU773, ICU400, and ICU399) from a tertiary-care hospital in Ho Chi Minh City. The isolates exhibited high resistance to a wide range of antibiotics, including carbapenems, aminoglycosides, and fluoroquinolones, while maintaining susceptibility to colistin. Whole-genome sequencing was used to analyze the genomic profiles, resistance genes, and sequence types of the isolates. All three isolates possessed *blaOXA-23* and additional resistance genes such as *blaADC* and aminoglycoside-modifying enzymes (AMEs). MLST analysis revealed distinct genetic lineages, with ICU773 and ICU400 belonging to Sequence Types 2/195 and 2/Novel and ICU399 to Sequence Types 571/804. In silico analysis further identified several efflux pump genes and other resistance mechanisms, including the presence of the *adeABC, adeFGH*, and *AbaQ* pumps. These findings highlight the complexity of CRAB's genomic diversity and resistance mechanisms in the region, underscoring the urgent need for continuous surveillance and novel therapeutic strategies to combat this growing healthcare threat.

## 1. Introduction

*Acinetobacter baumannii* (*A. baumannii*) is a species of bacteria that is an opportunistic pathogen and causes a variety of different types of infections [1]. The high risk of *A. baumannii* infection includes hospital patients and long-term care facility residents, especially patients who received complex medical care, including intensive care unit (ICU) admission or having invasive devices, severe or chronic wounds, recently taken antibiotics, and were admitted to the same room or unit as a person colonized or infected with *A. baumannii* [2]. Among multidrug-resistant (MDR) bacteria, carbapenem-resistant *A. baumannii* (CRAB) is a major concern due to the limited therapeutic options [3].

Infections caused by CRAB do not respond to common antibiotics, and some CRAB are resistant to all available antibiotics. CRAB spreads through direct and indirect contact with patients infected or colonized with CRAB or contaminated environments, surfaces, and equipment [4]. Carbapenem resistance in *A. baumannii* is usually the result of the expression of an OXA-type *β* or occasionally metallo-*β*-lactamases such as the IMP, VIM, and NDM groups. The acquired OXA-type *β*-lactamases in *A. baumannii* are encoded by genes belonging to five main groups—*oxa23* (or *blaOXA-23*-like), *oxa40* (or *blaOXA-40*-like), *oxa58* (or *blaOXA-58*-like), *oxa134* (or *blaOXA-134*-like), and *oxa143* (or *blaOXA-143*-like). The most common of these resistance mechanisms globally is *oxa23* [5]. In Vietnam and other countries in the region, *oxa23* is dominant; it can be found with frequencies greater than 70% [6]. In *A. baumannii*, *oxa23* is located within a transposon mobilized by one or more insertion sequences, which have enabled the resistance genes to be widespread to many different plasmids and lineages within the species [7].

The majority of *A. baumannii* isolates belong to one of eight international clonal complexes (ICCs), which correspond to specific multilocus sequence typing (MLST) sequence types (STs) and clonal complexes (CCs) [8]. There are two MLST schemes for *A. baumannii*—the Pasteur scheme (B) and the Oxford scheme (O), with the Pasteur scheme containing genes that are less prone to recombination than those in the Oxford scheme [8]. In the South East Asia region, ST2 has been frequently reported as the major ST of *A. baumannii* [9–14]. CCs such as CC1, CC2/CC92 (Pasteur/Oxford scheme), and CC4 are commonly found across this region, with CC1 being prominent in some regions like Taiwan and CC2 being more widespread in countries like Pakistan [15]. The distribution of STs and CCs varies significantly between countries, with certain clones like ST2(B) and CC2 being highly prevalent in hospital-acquired infections, especially those resistant to carbapenems [6].

In Vietnam, *A. baumannii* has emerged as a predominant etiological agent of healthcare-associated infections (HAIs), contributing to a substantial healthcare burden [16]. Epidemiological data from various studies conducted across the country have revealed a significant rise in CRAB isolates since 2009, particularly in association with ventilator-associated pneumonia (VAP) and bloodstream infections (BSIs) [17]. The prevalence of CRAB strains has been reported to range from 55% to 90%, with mortality rates in VAP patients at tertiary hospitals reaching as high as 52%. Extensively drug-resistant (XDR) *A. baumannii* strains are also prevalent; for instance, 90% of *A. baumannii* isolates from five medical centers in Vietnam were found to exhibit resistance to multiple antimicrobial classes, including *β*-lactams, cephalosporins, aminoglycosides, and carbapenems [12]. Carbapenem resistance in *A. baumannii* is primarily attributed to the presence of the *blaOXA-23* gene, while the *blaNDM-1* gene has been detected sporadically. The absence of a vaccine for *A. baumannii* and the increasing resistance to carbapenems and other last-line antibiotics highlight the urgent need for continuous surveillance of CRAB's epidemiology and its clinical impact in Vietnam. The objective of this study was to investigate the clinical characteristics and genomic epidemiology of CRAB isolates from a tertiary-care hospitals in Ho Chi Minh City, Vietnam.

## 2. Materials and Methods

### 2.1. Clinical Isolates

Three clinical isolates of *A. baumannii*—designated ICU773, ICU400, and ICU399—were collected from patients admitted to the ICU at Cho Ray Hospital during the period of January 1, 2020, to June 1, 2020. The isolates were obtained from quantitative sputum cultures processed in the Department of Clinical Microbiology at Cho Ray Hospital, Ho Chi Minh City, Vietnam. The clinical profiles of the patients from whom these isolates were derived as follows: ICU399 was recovered from a patient diagnosed with septic shock secondary to a biliary tract infection caused by choledocholithiasis. The patient had a history of endoscopic retrograde cholangiopancreatography (ERCP) and biliary stent placement and subsequently developed multiple organ failure and pneumonia. ICU400 was isolated from a patient presenting with septic shock, suspected to have originated from a urinary tract infection complicated by pyelonephritis and a left renal abscess. Finally, ICU773 was obtained from a postoperative patient recovering from surgical intervention for traumatic injuries sustained in a traffic accident.

### 2.2. Microbiological Culture and Antibiotic Susceptibility Testing (AST)

Sputum culture was performed on fresh sheep blood, MacConkey, and chocolate agars. Bacterial identification and AST were performed using the VITEK 2 Compact system (bioMérieux, France), an automated platform for microbial identification and antimicrobial resistance (AMR) profiling. Minimum inhibitory concentration (MIC) values and AMR categorizations were interpreted in accordance with the Clinical and Laboratory Standards Institute (CLSI) guidelines (CLSI, 2020). The analysis encompassed 12 antibiotics from various pharmacological classes, including *β*-lactams, aminoglycosides, fluoroquinolones, and polymyxins.

### 2.3. Sample Preparation and Whole Genomic Sequencing

Genomic DNA was extracted from bacterial cultures using the DNeasy Blood & Tissue Kit (QIAGEN, Hilden, Germany) according to the manufacturer's protocol. The DNA concentrations were quantified with a Qubit Fluorometer (Thermo Fisher Scientific, Waltham, Massachusetts, United States) to ensure precise measurements for subsequent library preparation. DNA fragmentation and adapter ligation were carried out using the Nextera DNA Flex Library Prep Kit (Illumina, San Diego, California, United States), following the manufacturer's guidelines, to generate DNA fragments of 200–600 base pairs, suitable for sequencing on the iSeq 100 System (Illumina, San Diego, California, United States).

### 2.4. Gene Content Analysis and Species Identification

The quality of raw sequencing reads was evaluated using FASTQC (v0.12.1; https://github.com/s-andrews/FastQC). Reads with low quality scores (*Q* < 15) and residual adapter sequences were trimmed using FASTP (v0.24.0) [18]. Following trimming, de novo assemblies were generated using Unicycler (v0.5.1) with the SPAdes assembly algorithm [19, 20]. Assembly quality and statistics were assessed with QUAST (v5.3.0), while bacterial genome annotation was performed using Prokka (v1.14.5) [21]. The completeness of draft genomes was evaluated with CheckM (v1.2.3) [22], and taxonomic classification was conducted using GTDB-Tk, a toolkit for genome taxonomy classification based on the Genome Taxonomy Databases [23]. The genome assemblies generated in this study have been deposited in the DDBJ database under BioProject Accession Number PRJDB19537 and are publicly available under the following accession numbers: BAAGBB010000001–BAAGBB010000013 for strain ICU399, BAAGBC010000001–BAAGBC010000009 for strain ICU400, and BAAGBD010000001–BAAGBD010000007 for strain ICU733. Capsule typing and MLST were performed using PathogenWatch (https://pathogen.watch), a web-based platform for the analysis of bacterial genomic data. The capsule type of each strain was identified using the capsule typing tool provided by PathogenWatch, which classifies strains based on the genetic determinants of capsule biosynthesis loci. Additionally, MLST was conducted using the pubMLST scheme available on the platform, which assigns STs based on the allelic profiles of housekeeping genes, allowing for the classification and comparison of bacterial strains. AMR and virulence genes were screened from assembled contigs using ABRICATE (v1.2.3), leveraging two key AMR databases: NCBI AMR and CARD [24] and VFDB database [25]. Prophage regions were predicted, and subsequent gene annotation was conducted using the PHASTEST web server [26].

### 2.5. The Antibiotic Resistance Profiling of Three MDR *A. baumannii* Strains

The MIC testing results indicate that the three MDR *A. baumannii* isolates (ICU773, ICU400, and ICU399) exhibited high levels of resistance to a broad range of antibiotics, including carbapenems, aminoglycosides, and fluoroquinolones (Table 1). This extensive resistance profile highlights significant therapeutic challenges posed by these strains. All isolates showed resistance to *β*-lactam antibiotics, with elevated MICs for ticarcillin (≥ 128 *μ*g/mL), ticarcillin/clavulanic acid (≥128 *μ*g/mL), piperacillin (≥128 *μ*g/mL), piperacillin/tazobactam (≥128 *μ*g/mL), ceftazidime (≥64 *μ*g/mL), and cefepime (≥64 *μ*g/mL), indicating widespread resistance across this class. Furthermore, the isolates displayed resistance to carbapenems, such as imipenem and meropenem (MICs ≥ 16* μ*g/mL), reducing susceptibility to this critical last-resort antibiotic class. Resistance to aminoglycosides was also observed, with high MICs for gentamicin (≥ 16 *μ*g/mL) and tobramycin (≥ 16 *μ*g/mL) (Table 1).

Additionally, three isolates exhibited resistance to fluoroquinolones, as evidenced by elevated MICs for levofloxacin (≥ 8 *μ*g/mL) across the board. Although ciprofloxacin data was unavailable for isolate ICU773, isolates ICU400 and ICU399 showed resistance (MIC ≥ 4* μ*g/mL), suggesting fluoroquinolone resistance across all isolates. On the other hand, all isolates were susceptible to colistin (MIC ≤ 0.5* μ*g/mL), indicating it could still be an effective therapeutic option for these MDR strains. High resistance to trimethoprim/sulfamethoxazole (MIC ≥ 320 *μ*g/mL) was noted in all isolates, underscoring the extensive resistance profile. In contrast, variability in tetracycline resistance was observed, with isolates ICU773 and ICU400 showing resistance to doxycycline, while ICU399 was susceptible (Table 1).

### 2.6. Assembly Metrics, Completeness Assessment, and MLST of Three *A. baumannii* Isolates

The assembly metrics for the three isolates reveal genomic characteristics of *A. baumannii* species, although each assembly yields a slightly different number of contigs and total genome sizes. Isolate ICU773 has 79 contigs with an N50 of 167,723 bp and a total genome size of approximately 3.80 Mb, while isolate ICU400 consists of 98 contigs, an N50 of 90,123 bp, and a genome size of about 3.97 Mb. Isolate ICU399, with 132 contigs and an N50 of 103,749 bp, has a genome size of around 3.97 Mb. The GC content for all isolates is consistent at approximately 39%, aligning with typical *A. baumannii* genomic profiles. These assembly metrics suggest a high-quality assembly with sufficient completeness, enabling reliable downstream analysis of genetic features and comparative genomics among the isolates. The CheckM results provided display the quality assessment of genomic bins, specifically for bacterial lineage classification under the genus *Acinetobacter* (UID4685). All isolates belonged to the genus *Acinetobacter* lineage. Each bin was compared against 42 genomes, with 1193 markers identified and distributed across 254 marker sets. All bins showed completeness scores, with values of 99.98%, 99.95%, and 99.94%, indicating that the assembled contigs were nearly complete with minimal cross-contamination within the bins (Table 2). MLST was conducted using the pubMLST database. The Pasteur/Oxford scheme-based MLST analysis assigned isolates ICU733 and ICU400 to ST 2/195 and 2/Novel, while isolate ICU399 was classified as 571/804, reflecting distinct genetic lineages among the isolates (Table 2).

### 2.7. In Silico Analysis of AMR in Three *A. baumannii* Genomes

Our genomic analysis revealed several resistance genes and mutations, particularly in *β*-lactamase genes, which are capable of hydrolyzing compounds containing a *β*-lactam ring. The identified strains expressed genes encoding OXA-type carbapenemases, specifically *blaOXA-23* and *blaOXA-66*, alongside aminoglycoside-modifying enzymes (AMEs), consistent with observed high levels of resistance. *blaOXA-66*, part of the OXA-51 family, and *blaOXA-23* both contribute to carbapenem resistance in *A. baumannii*, posing a significant treatment challenge due to limited therapeutic options. Additionally, the class C *β*-lactamase genes *blaADC-198* and *blaADC-73*, associated with cephalosporin resistance, further complicate treatment options due to their role in enhancing carbapenem resistance (Table 3).

The primary mechanism of aminoglycoside resistance in *A. baumannii* is enzymatic modification via AMEs. Based on their mechanism of action, AMEs are classified into three groups: acetyltransferases, phosphotransferases, and nucleotidyltransferases. In this study, isolates ICU773 and ICU400 possessed three genes from the phosphotransferase class (*aph(6)-Id*, *aph(3*⁣^″^*)-Ib*, and *aph(3*⁣′*)-Ia*), while isolate AB399 harbored *ant(3*⁣^″^*)-IIa* from the nucleotidyltransferase class. Additionally, the presence of *ArmA*, a 16S rRNA methyltransferase, in isolates ICU773 and ICU399 confers high-level resistance to aminoglycosides.

*A. baumannii* also possesses multiple intrinsic resistance mechanisms, particularly through the expression of efflux pumps, which contribute to resistance against aminoglycosides, fluoroquinolones, and tigecycline. We identified genes associated with the activation of efflux pumps within the resistance–nodulation–division (RND) superfamily, the predominant efflux system in *A. baumannii*. Notably, this includes the *adeABC* pump complex, which plays a critical role in resistance to various antimicrobial agents, particularly aminoglycosides. The RND superfamily also includes the *adeFGH* and *adeIJK* efflux pumps, both associated with tigecycline resistance, which were detected in all three isolates (Table 3). Another efflux pump family present in *A. baumannii* is the multidrug and toxic compound extrusion (MATE) superfamily. Within this category, we identified the *AbeM* efflux pump across all three isolates, along with other pumps that conferred resistance to ciprofloxacin, gentamicin, meropenem, and imipenem (Table 3). The major facilitator superfamily (MFS) also plays a crucial role in *A. baumannii*'s antibiotic resistance. Specifically, the *AmvA* pump confers resistance to various classes of antibiotics, disinfectants, and dyes, while *AbaF* is associated with fosfomycin resistance. Additionally, all three isolates possessed a newly identified MFS efflux pump, *AbaQ*, which is the first MFS family member implicated in the export of quinolone-type antibiotics (Table 3).

Furthermore, the isolates harbored *msr(E)*, an ABC-F type ribosomal protection protein, and *mph(E)*, a macrolide 2⁣′-phosphotransferase, both of which confer resistance to macrolides, such as erythromycin, and streptogramin B antibiotics. Another resistant mechanism identified involved the alteration of dihydropteroate synthase (DHPS), the target enzyme of sulfonamides, which are commonly used in combination therapies. The modified DHPS enzymes encoded by *sul1* and *sul2* genes exhibit reduced binding affinity for sulfonamides, thereby conferring resistance. In this study, *sul1* was present in isolate ICU399, while *sul2* was found in isolates ICU400 and ICU773, respectively.

### 2.8. The Virulence Factor Profiling and Comparison Among Three *A. baumannii* Isolates

Three isolates possessed all key mechanisms for causing HAIs including outer membrane protein A (*ompA*), biofilm formation, capsule polysaccharide, phospholipase, and siderophore efflux system (Table S1). Two ST2 isolates (ICU400 and ICU773) possessed 119 and 121 virulence genes, while ST571 isolate (ICU399) possessed 105 of those. Together, these mechanisms enable *A. baumannii* to adhere, evade immune defenses, survive in hostile environments, and damage host tissues, making it a formidable pathogen in healthcare settings.

**Figure 1:**
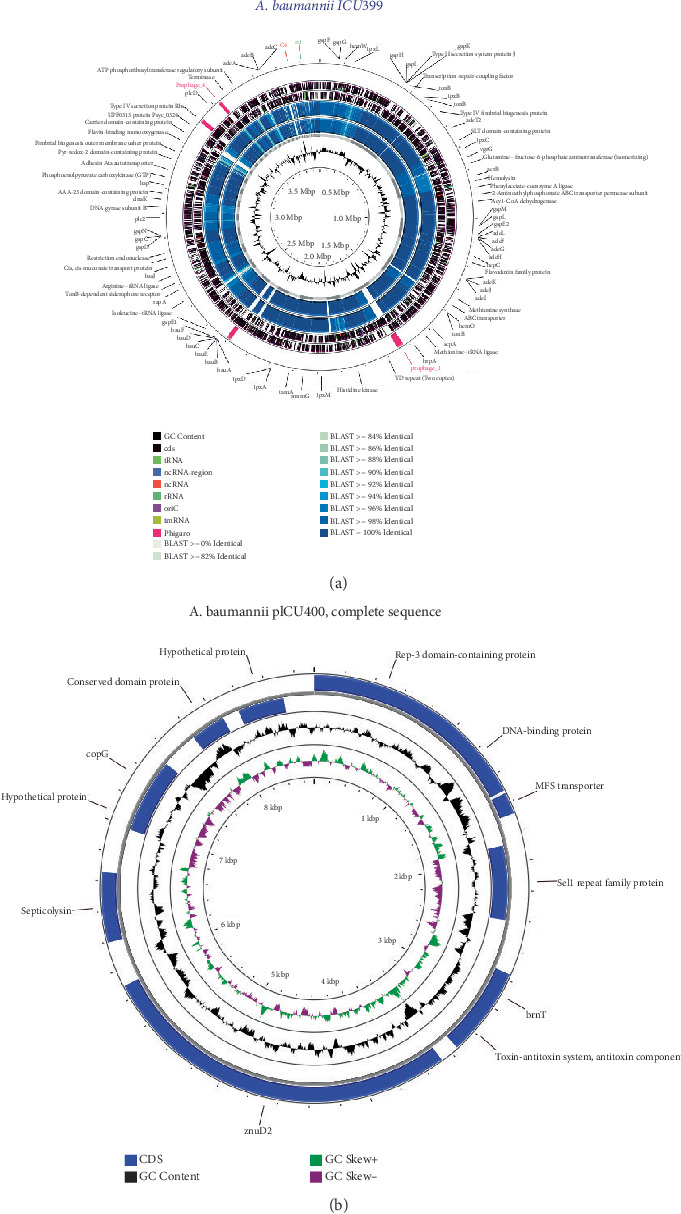
Comparative genomic analysis and plasmid map of *A. baumannii* isolates. (a) A comparative genomic analysis was conducted on three *A. baumannii* isolates: ICU399, ICU400, and ICU773. The innermost ring represented the GC% content of the assembled sequences from ICU399. The subsequent two rings displayed the BLAST alignment results of ICU400 and ICU773 against the ICU399 genome, with ICU399 serving as the query sequence. The next two layers depicted the coding sequences (CDSs) identified on both DNA strands of ICU399. Finally, the outermost ring highlighted prophage regions within the ICU399 genome as identified by Phigaro. (b) Genome map of *A. baumannii* pICU400 plasmid.

Figure 1 showed that all three isolates were highly similar in most of the genome, although there was a slight difference between the regions that resulted from gene transfer, including prophage regions (pink region). A comparative genomic analysis identified diverse prophage regions within the genomes of the three isolates. Both the ICU400 and ICU773 isolates contained prophage regions corresponding to *Psychrobacter* phage pOW20-A (NC_020841) and *Acinetobacter* phage YMC/09/02/B1251 (NC_019541). The ICU399 isolate harbored prophage regions associated with *Acinetobacter* phage YMC/09/02/B1251 (NC_019541), *Enterobacteria* phage mEp235, and *Bordetella* phage BPP-1 (Table S2). We recovered the circular contig, which was assigned to a novel plasmid in ICU773 and ICU400 (pICU400), similar to the plasmid p1OC081 (NZ_CP087306) from *A. baumannii* strain OC081 (Figure 1).

## 3. Discussion

The findings of this study underscore the alarming MDR profiles of *A. baumannii* isolates obtained from ICU settings, highlighting the significant therapeutic challenges posed by infections caused by this pathogen. Notably, *A. baumannii* has also been classified as a One Health ESKAPE pathogen, given its detection in animals, food sources, and environmental reservoirs, thus raising concerns about its zoonotic potential and environmental persistence. In our study, all three isolates—ICU773, ICU400, and ICU399—exhibited high-level resistance to *β*-lactams, carbapenems, aminoglycosides, and fluoroquinolones, with susceptibility retained only to colistin. This resistance pattern is consistent with global trends observed in MDR and XDR *A. baumannii* strains, particularly in developing countries where infection control measures and antibiotic stewardship programs may be limited. This extensive resistance profile emphasizes the urgent need for alternative treatment strategies and underscores the critical importance of ongoing AMR surveillance in clinical environments.

Through MLST analysis, we identified STs ST2 and ST571 among the isolates. These STs are globally prevalent and strongly associated with carbapenem resistance, particularly in *A. baumannii* strains involved in hospital-associated outbreaks. Both ST2 and ST571 have been identified as dominant CRAB lineages in multiple regions, including Vietnam, and exhibit distinct AMR gene profiles, which correlate with the extensive resistance patterns observed in the current isolates. These lineages are known to carry a diverse array of resistance genes, including *blaOXA-23*, *blaADC*, and AMEs, which collectively contribute to the extensive MDR phenotypes observed. The presence of efflux pump systems such as *adeABC*, *adeFGH*, and *AbaQ* further complicates treatment, as they enhance resistance to multiple drug classes and reduce intracellular drug concentrations. Targeting efflux pump systems represents a promising therapeutic approach. It suggests that interfering with efflux regulation—rather than efflux activity directly—may provide a novel strategy to overcome MDR phenotypes without incurring toxicity from traditional efflux inhibitors. These insights pave the way for adjunct therapies aimed at resensitizing resistant strains to conventional antibiotics.

Despite the high level of resistance, all isolates remained susceptible to colistin, supporting its continued use as a last-line treatment. However, the emergence of colistin resistance and associated nephrotoxicity necessitates cautious and well-regulated application. Additionally, the limited availability of rapid and accurate diagnostic tools in clinical settings presents a challenge in correctly identifying *A. baumannii*, potentially delaying appropriate therapy. This issue has been documented in recent literature, emphasizing the importance of improving diagnostic accuracy.

A perplexing observation in our study was the presence of carbapenem resistance in isolates despite the restricted use of imipenem and meropenem in Vietnam's public health system. This phenomenon may be attributed to horizontal gene transfer from environmental or agricultural sources, the importation of resistant clones, or unregulated antibiotic use in nonhospital settings. The potential for resistant genes to be carried on mobile genetic elements such as plasmids and integrons further exacerbates the spread across different bacterial populations and environments.

Our study is not without limitations. First, the small sample size limits the generalizability of the findings and precludes a comprehensive understanding of CRAB transmission dynamics within the ICU. Furthermore, the use of short-read sequencing constrained our ability to resolve complete genomic structures and identify the precise locations of resistance determinants, especially those harbored on plasmids or transposons. Future studies leveraging long-read sequencing technologies will be critical to fully elucidate the genomic architecture and mobilome of *A. baumannii*, thereby advancing the development of targeted interventions.

In conclusion, this study provides valuable insight into the genomic and resistance profiles of MDR *A. baumannii* isolates in a Vietnamese ICU, reinforcing the urgent need for enhanced diagnostic capabilities, novel therapeutic approaches such as efflux regulator inhibitors, and international collaboration to address the growing threat posed by ESKAPE pathogens in both clinical and community settings.

## Figures and Tables

**Table 1:** Antibiotic resistance profiles of three *A. baumannii* isolates (ICU773, ICU400, and ICU399).

**Antibiotics**	**MIC (mg/L)**		
	**ICU773**	**ICU400**	**ICU399**
Ticarcillin	≥ 128 (R)	≥ 128 (R)	N.A.
Ticarcillin/clavulanic acid	≥ 128 (R)	≥ 128 (R)	≥ 128 (R)
Piperacillin	≥ 128 (R)	≥ 128 (R)	≥ 128 (R)
Piperacillin/tazibactam	≥ 128 (R)	≥ 128 (R)	≥ 128 (R)
Ceftazidime	≥ 64 (R)	≥ 64 (R)	≥ 64 (R)
Cefepime	≥ 64 (R)	≥ 64 (R)	≥ 64 (R)
Imipenem	≥ 16 (R)	≥ 16 (R)	≥ 16 (R)
Meropenem	≥ 16 (R)	≥ 16 (R)	≥ 16 (R)
Gentamicin	≥ 16 (R)	≥ 16 (R)	≥ 16 (R)
Tobramycin	≥ 16 (R)	≥ 16 (R)	≥ 16 (R)
Ciprofloxacin	N.A.	≥ 4 (R)	≥ 4 (R)
Levofloxacin	≥ 8 (R)	≥ 8 (R)	≥ 8 (R)
Colistin	≤ 0.5 (S)	≤ 0.5 (S)	≤ 0.5 (S)
Trimethoprim/sulfamethoxazole	≥ 320 (R)	≥ 320 (R)	≤ 20 (S)
Doxycycline	R	R	S

**Table 2:** Genome characteristics of three *A. baumannii* isolates from ICU patients.

	**ICU399**	**ICU733**	**ICU400**
Number of contigs	132	79	98
Total contig length (bp)	3,979,868	3,904,623	3,870,473
Max contig (bp)	247,304	292,477	220,363
Mean contig (bp)	30,150	49,425	39,494
Median contig (bp)	5165	11,208	22,633
N50	103,749	167,723	90,123
L50	13	10	14
GC%	38.87	38.93	38.91
Completeness (%)	99.95	99.98	99.94
Contamination (%)	0.52	0.63	0.63
Sequence type (pubMLST)			
Oxford	804	195	Novel
Pasteur	571	2	2
Surface polysaccharide typing	KL10	KL3	KL52
Number of CDS	3730	3660	3604
Number of rRNA	3	3	3
Number of tRNA	64	63	63
Novel plasmid	0	1	1

**Table 3:** Main mechanism conferred to AMR in three *A. baumannii* strains.

**Mechanism**		**ICU773**	**ICU400**	**ICU399**	**Predicted phenotype**
*β*-Lactamase	Class A	*TEM-12*	*TEM-12*	*TEM-12*	Cephalosporin
	Class B	NA	NA	NA	
	Class C	*blaADC-73*	*blaADC-73*	*blaADC-198*	Cephalosporin
	Class D	*blaOXA-23*	*blaOXA-23*	*blaOXA-23*	Carpapenem
		*blaOXA-66*	*blaOXA-66*	*blaOXA-66*	Carpapenem

Enzyme modification	Acetyltransferases, phosphotransferases and nucleotidyl transferases of aminoglycosides	*aph(6)-Id*, *aph(3*⁣^″^*)-Ib*, *aph(3*⁣′*)-Ia*	*aph(6)-Id*, *aph(3*⁣^″^*)-Ib*, *aph(3*⁣′*)-Ia*	*ant(3*⁣^″^*)-IIa*,	Spectinomycin, streptomycin, and kanamycin
	Methylate the 16S rRNA within the bacterial 30S ribosomal subunit of 16S rRNA	*ArmA*	*ArmA*	*ArmA*	Gentamicin

Nonenzymatic mechanisms	Activation of the efflux pumps	*adeA*, *adeB*, *adeC*, *adeL*, *abeM*, *abeN*, *adeF*, *adeG*, *adeH*, *adeK*, *adeJ*, *adeI*	*adeA*, *adeB*, *adeC*, *adeL*, *abeM*, *abeN*, *adeF*, *adeG*, *adeH*, *adeK*, *adeJ*, *adeI*	*adeA*, *adeB*, *adeC*, *adeL*, *abeM*, *abeN*, *adeF*, *adeG*, *adeH*, *adeK*, *adeJ*, *adeI*	
	Tetracycline efflux protein	*tet(B)*	*tet(B)*	N.A.	Tetracycline
	MFS superfamily	*AbaQ*, *AmvA*, *AbaF*	*AbaQ*, *AmvA*, *AbaF*	*AbaQ*, *AmvA*, *AbaF*	

Protein synthesis	Ribosomal protection	*msr(E)*, *mph(E)*	*msr(E)*, *mph(E)*	*msr(E)*, *mph(E)*	Macrolide
	Target modification	*sul2*	*sul2*	*sul1*	Sulfonamide

## Data Availability

The data that support the findings of this study are available from the corresponding author upon reasonable request.

## Supporting Information

Supporting Information Additional supporting information can be found online in the Supporting Information section. Table S1: Virulence factors predicted in three *Acinetobacter baumannii* isolates. Table S2: Predicted prophage regions in the ICU773 isolate.
